# Enhanced Performance of Community Health Service Centers during Medical Reforms in Pudong New District of Shanghai, China: A Longitudinal Survey

**DOI:** 10.1371/journal.pone.0125469

**Published:** 2015-05-07

**Authors:** Xiaoming Sun, Yanting Li, Shanshan Liu, Jiquan Lou, Ye Ding, Hong Liang, Jianjun Gu, Yuan Jing, Hua Fu, Yimin Zhang

**Affiliations:** 1 School of Public Health, Public Health Security Key Laboratory of Ministry of Education, Fudan University, Shanghai, China; 2 Pudong Institute for Health Development, Shanghai, China; 3 Health and Family Planning Commission of Pudong new district, Shanghai, China; 4 School of Social Development and Public Policy, Fudan University, Shanghai, China; Old Dominion University, UNITED STATES

## Abstract

**Background:**

The performance of community health service centers (CHSCs) has not been well monitored and analysed since China’s latest community health reforms in 2009. The aim of the current investigation was to evaluate the performing trends of the CHSCs and to analyze the main factors that could affect the performance in Pudong new district of Shanghai, China.

**Methods:**

A regional performance assessment indicator system was applied to the evaluation of Pudong CHSCs’ performance from 2011 to 2013. All of the data were sorted out by a panel, and analyzed using descriptive statistics and a generalized estimating equation model.

**Results:**

We found that the overall performance increased annually, with a growing number of CHSCs achieving high scores. Significant differences were observed in institutional management, public health services, basic medical services and comprehensive satisfaction during the period of three years. However, we found no differences in the service scores of Chinese traditional medicine (CTM). The investigation also demonstrated that the key factors affecting performance were the location, information system level, family GP program and medical association program rather than the size of the center. However, the medical association participation appeared to have a significant negative effect on performance.

**Conclusions:**

It can be concluded from the three-year investigation that the overall performance was improved, but that it could have been further enhanced, especially in institutional management and basic medical service; therefore, it is imperative that CHSCs undertake approaches such as optimizing the resource allocation and utilization, reinforcing the establishment of the information system level, extending the family GP program to more local communities, and promoting the medical association initiative.

## Introduction

In China, the community health service (CHS) was launched in the 1990s. In the past 20 years, the central government has issued a series of significant health policies to promote the reforms of community health service centers (CHSCs) and enhance the primary health care system. To improve the primary health service system was one of the five key initiatives in the latest round of medical reforms initiated in 2009, with a view to facilitating access to the national health services by regulating and unifying the medical practices [1]. However, the actual performance of CHS reforms remains unknown. Thus, to improve the efficacy and quality of CHS, there is a great need of a scientific appraisal mechanism of performance-management, which is an important tool for process control, performance evaluation and problem correction in implementing the CHS policies and measuring the appropriateness and rationality of CHS planning. Moreover, the mechanism can function to provide some theoretical and technical support for the further development of CHS [2].

From research perspectives, the performance appraisal on CHS can be manipulated both macroscopically and microscopically [3,4], laying a particular emphasis on benefit evaluation and regional resource allocation, and providing comparisons of CHSCs’ operating effect and budget allotment. To address the requirements needs a scientific and appropriate appraisal system [5–7]. The Canadian Institute for Health Information (CIHI) once established the indicators system for primary health care, covering such aspects as service comprehensiveness and accessibility, quality improvement and patient-centered care [8], while the Royal Australian College of General Practitioners (RACGP) released a GP standard treatment system in 1996, including elements of GP treatment service, patients’ rights and needs, quality improvement, education and environment factors, to highlight accessibility and effectiveness, technical and allocation efficiency, service appropriateness, patients’ satisfaction, and evaluation on continuous care [9]. Furthermore, the Quality and Outcome Framework (QOF), a thousand-point management model in the UK, was introduced as a part of the General Medical Services Contract in 2004, so as to standardize the improvements in clinical care, organization management, patient experience and alternative services [10]. To some extent, these pioneering models are well worth borrowing, but we came to realize that different stages of CHS development and cultural differences in medical management may require a specific and unique appraisal system.

Since 2002 the nation-wide CHS appraisals have been reported to be mainly of comparative and descriptive studies using measurement tools like Accumulative Synthetical Scoring (ASS), Rank Sum Ratio (RSR), Technique for Order Preference by Similarity to Ideal Solution (TOPSIS), Fuzzy Comprehensive Evaluation (FUZZY) and Analytic Hierarchy Process (AHP) [11–13]. Most of the studies focused on evaluating regional CHS systems from a macroscopic viewpoint, and discussing the framework guidance covering government support, financial funding, institution establishment, service contents and process, health outcomes and satisfaction, etc. [5,12,14]. However, a multi-factor statistical analysis hasn’t been reported on the factors that influence the outcomes in the literature. Although there have been a few investigations evaluating CHSCs [15, 16], little valuable information could be obtained from the literature. Moreover, such microscopic studies seemed to be obsolete because they were conducted before 2009. Therefore, it is necessary that such researches be updated and deepened. The former Ministry of Health (MOH) of China issued new guidelines on the National Performance Assessment Indicators System of Community Health Service Institutions (NPAISCHSI) in 2011 [17]. However, given the huge differences in the socio-economic development between mid-western and eastern China and the big variations in the progress of community health reforms, a system of unified standards could hardly apply to equitable evaluation in all areas. Therefore, it is important that the national guidelines be adapted and modified.

Pudong new district (Pudong), located in the east of Shanghai, covers 1290.63 km^2^, with a population of 5.42 million inhabitants (2013) in over 38 communities. Symbolic of reform and opening-up in China, Pudong is far ahead of other domestic areas in terms of prosperity and CHS. In all the state-owned CHSCs have been conducted the Essential Medicine System (EMS), the Basic Public Health Services Equalization (BPHSE), the Revenue and Expenditure Separate Management (RESM) mechanism and the implementation of the informatization standard configuration, and also into them since 2011 have been introduced the family GP program and the medical association initiative.

The current investigation was of a 3-year longitudinal study in Pudong, which continuously measured the performance of the state-owned CHSCs using a system of appropriate appraisal indictors, evaluated the reforming trends of CHSCs, and made comparisons of performance differences among the appraisal dimensions, and further analyzed the main factors that could affect the performance of the CHSCs, the aim of which was to provide the heath administrators and managers with microscopic decision-making information, as well as methodology references for researches in other similar regions.

## Materials and Methods

### Ethics Statement

The survey participants of our study were all state-owned CHSCs involved in Pudong, which were Bei-cai CHSC, Jin-yang CHSC, Zhou-jiadu CHSC, Da-tuan CHSC, San-lin CHSC, Shang-gang CHSC, Pu-xing CHSC, Hu-dong CHSC, Chuan-sha CHSC, Huang-lou CHSC, Lu-jiazui CHSC, Wei-fang CHSC, Zhu-qiao CHSC, Dong-ming CHSC, Hua-mu CHSC,Ying-bo CHSC, Hui-nan CHSC, Nan-matou CHSC, Xin-chang CHSC, Lian-yang CHSC, Cao-lu CHSC, Tang-qiao CHSC, Gao-qiao CHSC, Gao-hang CHSC, Ni-cheng CHSC, Zhou-pu CHSC, He-qing CHSC, Shu-yuan CHSC, Hang-tou CHSC, Jin-qiao CHSC, Kang-qiao CHSC, Xuan-qiao CHSC, Gao-dong CHSC, Zhang-jiang CHSC, Liu-zao CHSC, Tang-zhen CHSC, Ji-chang CHSC, Lao-gang CHSC, Wan-xiang CHSC, Yang-jin CHSC, Sun-qiao CHSC, Lu-chaogang CHSC, Wang-gang CHSC, Ling-qiao CHSC, 44 in 2011 and 2012 year, Jiang-zhen CHSC added with 45 in 2013 year, respectively. The study did not involve any ethical issues. And our investigations were permitted by every CHSC, whose legal representatives, without exception, expressed informed consent and guaranteed the verification and validation of the survey data signing the commitment files. Also the data were obtained only for academic research purposes. All information obtained was anonymized and de-identified prior to analysis.

### Measuring instrument

#### Basic sociological information

The sociological characteristics of each CHSC were mainly composed of seven variables: regional location, informatization level, involvement in the family GP program, participation in medical association, the number of sick beds, the number of employees and the floor area of the buildings. The regional location fell into three categories: urban division, suburban district and rural area, which were ascertained by population density (residents per km^2^) and socio-economic development level (GDP per capita) of the area in which the center is located. A urban division required both population density and socio-economic development level above the average, while a suburban district has only one of them above the average, and a rural area has neither of them above the average. The informatization level was divided into three grades: high, medium and low, assessed against a standard configuration inside the center. Thus those which possessed self-developed systems were considered as high-grade centers, and those with standard configurations, as medium, while those without the standard configuration, as low.

#### Performance assessment system

Based on NPAISCHSI issued by the MOH in June 2011, the performance assessment indicators system and scoring criteria for the CHSCs were drafted according to the local health practice and the recent reform policies, with the assistance of the national professionals. The quality and equity of the system was further improved by pre-trialing the localized protocol involving three CHSCs in different regions, and well affirmed by the health administrative departments and assessment institutes. Thus, the revised index of performance assessment system of CHSCs in Pudong new area was comprised of five dimensions, which were institutional management, public health service, basic medical service, CTM service, and comprehensive satisfaction. Two-thirds of the indicators in the index system conformed to the national standards, while the rest agreed with the district standards, which accounted for 1200 points in total. Moreover, the scores were approximately the same between the pilot and formal appraisal on the three centers (margin of error < 0.17) in the first year of 2011, which reflected the validity and reliability of the measurement.

The assessment of institutional management performance in Part 1 covered 14 second-level and 44 third-level indicators, with a total score of 410. The second-level indicators accounted for 18 points for the center’s environment, 14 for human resource management, 14 for continuing education, 15 for academic research, 28 for financial and asset management, 18 for drug administration, 12 for rural health management, 18 for rural cooperative medical care, 28 for progress in spiritual civilization, 10 for information management, 118 for service mode, 100 for operating efficiency, 17 for rectification follow-up, and punitive points for the safety management.

Public health service performance was estimated by 1 second-level indicator and 16 third-level indicators in Part 2, with a total score of 350. This second-level indicator chiefly focused on the quality and quantity management of BPHSE items. The third-level indicators consisted of child health care (age: 0–6 years) (33), preventive vaccination (42.6), school healthcare (22.2), occupational groups health care (3.8), women health care (34), elderly healthcare (13.1), prevention and control of chronic diseases (40), mental health (19.2), disposal of infectious diseases and public health emergencies (37.2), health supervision and coordination (19.2), management of disease and related factors (14), health education (19.2), vital statistics (2.5), Elderly medical (30), health records (20), and punitive points for major events of public health.

Performance evaluation of the basic medical service in Part 3 covered 12 second-level and 29 third-level indicators, scoring 240. They referred to medical service ability, health insurance management and rehabilitation service, accounting for the scores of 56, 35 and 30, respectively, while quality control of medical practice, rational use of drugs, quality control for nursing, nosocomial infection management, management by law scored 24, 20, 15, 11, 9points, respectively; and another 4 items in clinical laboratory quality control, radiation, electrocardiogram and B-ultrasonic wave quality control, and health care costs were each allocated 10 points.

In Part 4, the performance evaluation on the CTM service consisted of 2 second-level and 17 third-level indicators, with a total score of 100. The second-level indicators referred to CTM service number, the quality of CTM service, allocated 40 and 60 points, respectively.

In Part 5, a comprehensive measure of satisfaction was assessed through 3 second-level and 3 third-level indicators, with a total score of 100. Overall patient satisfaction with the CHSCs, which accounted for 60 points, was measured in line with safety issues, economy of medical services, comfort and convenience experience, as well as effectiveness, by the sampled patients seeking treatment. Overall staff satisfaction with the CHSCs, allocated another 40 points, was surveyed in a wide range of aspects comprising working environment, income level, training accessibility, promotion opportunity and career prospect. Criticism was given punitive points, including the policy criticism and service quality criticism. Recommended by the MOH, the satisfaction questionnaires were used based on the national standard surveys for the patient and physician, and verified as valid.

Furthermore, the scoring criterion of each third-level indicator was rationally set up in advance concerning the examination method of the MOH and the local features. A total score was derived from the combined scores of the different performance dimensions. Then, a summary calculation of the five dimensions was recorded as an absolute value of overall performance for each center, and finally a relative score scale (ratio) was acquired through the absolute value divided by the total points (1200), in order to compare the CHSCs via a universal threshold. The relative overall performance was divided into 4 grades using a final score of ≥90% as excellent; 80–90% as good; 70–80% as medium; and 60–70% as poor.

### Data collection

A combination of quantitative and qualitative methodology was used to collect the data, the specific approaches as follows: Fifteen specialists from the administration departments of hospitals and universities were invited to engage in the current investigation, and they were equally divided into 5 groups to assess the performance in the five key dimensions mentioned above. A workshop was provided to ensure the smooth running of the evaluation process. All investigators declared that they had no competing interest with any center by signing an equity pledge and confidentiality agreement, and they were excluded from the data analysis to reduce any bias.

The data originated from three on-site sources: the center’s reported information, the review of the statistical documents and reports issued by the local health agencies and the survey of satisfaction questionnaires. In particular, the survey of patient satisfaction was anonymously and randomly conducted, while the investigation of staff satisfaction was anonymously performed with the whole staff.

All of the data was logically checked and sorted out by specific algorithms before further quantitative evaluations based on the criteria were made by a panel. Explanation was supplied or original documents were attached if some data was seen as equivocal. All assessment results were obtained under a careful inspection by the final researcher along with the specialists’ signatures to ensure the validity and fairness of the on-site evaluation.

### Statistical analyses

The data were analyzed using the statistical software package SPSS 18.0. The threshold of statistical significance was set at *P*<0.05 (two-tailed). Descriptive statistics (Mean±SD, percentage) was calculated to estimate the performance distribution of the CHSCs, and a scoring ratio (%) was obtained by dividing the total points. After that, the generalized estimating equation (GEE) for the repeated measured data was obtained to examine whether the sociological characteristics of the CHSCs affected significantly their overall or dimension-specific performance. The independent variables contained year, regional location (X1), informationization level (X2), involvement in the family GP program (X3), medical association participation (X4), number of sick beds (X5), number of employees (X6), and floor area of the buildings (X7). Overall performance, institutional management, public health services, basic medical services, CTM services and comprehensive satisfaction were regarded as dependent variables, respectively. In the analysis of GEE, an identity function was employed as a link function, an unstructured model as a job related matrix, and the generalized estimating equation established as follows:

$$\begin{array}{l} Y_{ij}=\;\beta_{0}+\;\beta_{1}year1_{ij}+\;\beta_{2}year2_{ij}+\;\beta_{3}X11_{ij}+\;\beta_{4}X12_{ij}+\;\beta_{5}X21_{ij}+\;\beta_{6}X22_{ij}\\ +\;\beta_{7}X31_{ij}+\;\beta_{8}X32_{ij}+\;\beta_{9}X33_{ij}+\;\beta_{10}X34_{ij}+\;\beta_{11}X4_{ij}+\;\beta_{12}X5_{ij}+\;\beta_{13}X6_{ij}\\ +\;\beta_{14}X7_{ij}+\;\beta_{15}\left(year*X1\right)\;+\;\beta_{16}\left(year*X2\right)\;+\;\beta_{17}\left(year*X3\right)\;\\ +\;\beta_{18}\left(year*X4\right)\;+\;\beta_{19}\left(year*X5\right)\;+\;\beta_{20}\left(year*X6\right)\;+\;\beta_{21}\left(year*X7\right) \end{array}$$

## Results

### Sociological characteristics

From 2011 to 2013, it was observed that the CHSCs were evenly distributed in the urban divisions, suburban district and rural areas. In 2011 and 2012, more than 80% (36) of them possessed an informationization system at a medium level, while in 2013, 29.54% (13), one at a high level and 63.64% (28), one at a medium level. In 2011, only 13.64% (6) involved in the family GP program, but by 2012 and 2013, all extended the family GP program to three layers: demonstrating center, key center and standard center. Moreover, nearly 30.00% of them participated in the medical association program throughout the period of three years. The number of sick beds and employees and the floor area fluctuated within a narrow range from 2011 to 2013 (Table 1).

**Table 1:** Sociological characteristic of CHSCs in Pudong from 2011 to 2013.

Characteristics	2011	2012	2013
**Regional location N (%)**			
** Urban division**	14(31.82)	15(33.33)	15(34.09)
** Suburban district**	16(36.36)	15(33.33)	15(34.09)
** Rural area**	14(31.82)	15(33.33)	14(31.82)
**Level of informationization system N (%)**			
** High**	6(13.64)	6(13.33)	13(29.54)
** Medium**	36(81.82)	36(80.00)	28(63.64)
** Low**	2(4.54)	3(6.67)	3(6.82)
**Family GP program N (%)**			
		DC*2(4.44)	DC*2(4.54)
** Yes**	6(13.64)	KC*10(22.22)	KC*10(22.73)
		SC*33(73.33)	SC*32(72.73)
** No**	38(86.36)	—	—
**Medical association participation N (%)**			
** Yes**	13(29.55)	13(28.89)	12(27.27)
** No**	31(70.45)	32(71.11)	32(72.73)
**Number of beds (Mean±SD)**	80.48±9.23	59.53±39.20	60.61±38.15
**Number of employees (Mean±SD)**	140.82±13.14	137.96±43.58	144.20±43.99
**Building area (m** ^**2**^ **) (Mean±SD)**	7235.34±34.75	7266.20±2477.87	7007.55±2293.25

Note. DC* = Demonstrating center, KC* = Key center, SC* = Standard center.

### The trend of overall performance of CHSCs from 2011 to 2013

An increased overall percentage score, calculated from the various scores obtained at each CHSC, ranged from 66.82 to 96.86, with an overall performance mean of 78.53±6.11, 82.58±5.66, and 87.70±5.02, respectively, on which the performance was classified as excellent, good, medium or poor (Table 2). In 2011, 21 centers (47.73%) were defined as medium, as indicated by the scores of 70–80; in 2012, 28 (62.22%) centers, as good by the scores of 80–90; in 2013, 14 and 28 centers, as excellent and good, with no one as poor. Moreover, it was found that the mean score per grade had been improved by 1–2 points in comparison with that in the previous year (Table 2).

**Table 2:** The distribution of overall percentage in CHSCs from 2011 to 2013.

Group	Percentage range	2011		2012		2013	
		N (%)	Mean±SD	N (%)	Mean±SD	N (%)	Mean±SD
**Excellent**	≥90	1(2.27)	90.85±0.00	3(6.67)	92.47±2.16	14(31.82)	93.22±2.29
**Good**	[80–90)	18(40.90)	84.02±2.85	28(62.22)	84.80±2.95	28(63.64)	85.67±3.12
**Medium**	[70–80)	21(47.73)	75.20±2.35	12(26.67)	77.16±1.29	2(4.55)	77.55±2.20
**Poor**	[60–70)	4(9.10)	68.25±1.18	2(4.44)	69.15±1.12	0(0.00)	—
**Total**	[60–100]	44(100.00)	78.53±6.11	45(100.00)	82.58±5.66	44(100.00)	87.70±5.02

### Statistical description and analysis on various dimensions of performance

As indicated by the mean scores of each dimension of performance (Table 3), we found that in 2011 the best performance was achieved in the services of public health and CTM, and that in 2012 quick advances, in the performance of basic medical services, while the comprehensive satisfaction performed poorly. In all 5 dimensions, most of the performance scores were higher than in 2013 than in the other two respective years, especially in the public health services and comprehensive satisfaction. Furthermore, we found that these differences were significant in the overall performance, institutional management, public health services, basic medical services and comprehensive satisfaction scores over the three years. However, no differences were found in the CTM services during the three years.

**Table 3:** Performance comparisons in different dimensions of CHSCs from 2011 to 2013.

Performance dimensions	2011		2012		2013		F	*P* *
	Mean±SD	%	Mean±SD	%	Mean±SD	%		
**Institutional management**	287.65±46.07	68.49	314.56±30.37	74.90	323.25±31.00	78.84	11.39	<0.001
**Public health services**	311.81±19.39	89.09	307.51±17.02	87.86	319.74±12.22	91.35	6.28	0.003
**Basic medical services**	146.07±15.17	63.51	192.45±19.57	83.67	195.50±15.24	81.46	119.84	<0.001
**CTM services**	87.80±7.05	87.80	85.55±10.93	85.55	87.69±7.24	87.69	0.97	0.383
**Comprehensive satisfaction**	76.32±10.07	76.32	67.45±9.77	67.45	95.53±5.88	95.53	118.26	<0.001
**Overall performance**	909.65±68.11	75.80	980.42±70.20	81.70	041.97±60.95	86.83	43.53	<0.001

Note. *significant at the 0.05 level (2-tailed).

### Generalized linear model analysis of the influential factors on CHSC performance

The results demonstrated that the variables of year, regional location, level of informationization system, family GP program, medical association participation, number of employees and floor area of the buildings contributed significantly to overall performance (*P*<0.05). Moreover, the year, regional location, level of informationization system, family GP program, number of employees and floor area of the buildings were shown to be positive predictor variables to overall performance, with medical association participation as a negative predictor variable. Whereas the statistical interaction were also observed between year and informationization level, year and number of employees, as well as year and floor area of the buildings in the model of overall performance.

In the model of institutional management performance, the variables of year, regional location, level of informationization system, family GP program, medical association participation and number of employees exerted a significant impact on institutional management performance, including the year, regional location, level of informationization system, family GP program and number of employees as positive predictor variables and medical association participation as a negative predictor. However, the interaction (Year*X7) was significant (*P*<0.05).

In the model of public health services performance, the year, level of informationization system and medical association participation were the main influencing factors. Still, medical association participation was the negative predictor variable (Table 4).

**Table 4:** Analysis of influencing factors on overall performance, institutional management, and public health services performance.

Independent variables		Overall performance^①^			Institutional management^②^			Public health services		
		β	SE	*P* *	β	SE	*P* *	β	SE	*P* *
**Intercept**		67.35	2.53	**<0.001**	272.35	20.94	**<0.001**	293.59	10.97	**<0.001**
**Year**	**2013y**	8.33	1.98	**<0.001**	-39.34	17.57	**0.025**	8.78	3.64	**0.016**
	**2012y**	4.93	3.49	0.158	-14.61	16.66	0.380	-2.84	3.66	0.439
	**2011y**	0.00^a^	-	-	0.00^a^	-	-	0.00^a^	-	-
**X1**	**Urban division**	3.00	0.94	**0.002**	8.99	8.23	0.275	-3.83	3.31	0.248
	**Suburban district**	2.88	0.70	**<0.001**	29.94	6.78	**<0.001**	-1.59	3.03	0.600
	**Rural area**	0.00^a^	-	-	0.00^a^	-	-	0.00^a^	-	-
**X2**	**High**	12.39	1.91	**<0.001**	52.54	7.17	**<0.001**	29.21	9.16	**0.001**
	**Medium**	10.96	1.60	**<0.001**	35.29	6.62	**<0.001**	23.10	8.73	**0.008**
	**Low**	0.00^a^	-	-	0.00^a^	-	-	0.00^a^	-	-
**X3**	**DC**	1.09	2.15	0.612	-11.64	12.84	0.364	1.61	6.59	0.807
	**KC**	2.73	0.95	**0.004**	14.10	6.37	**0.027**	-0.84	3.50	0.811
	**SC**	0.00^a^	-	-	0.00^a^	-	-	0.00^a^	-	-
	**Yes**	4.43	1.21	**<0.001**	32.98	7.89	**<0.001**	1.55	6.21	0.803
	**No**	0.00^a^	-	-	0.00^a^	-	-	0.00^a^	-	-
**X4**	**Yes**	-5.26	0.78	**<0.001**	-27.38	6.58	**<0.001**	-13.24	3.08	**<0.001**
	**No**	0.00^a^	-	-	0.00^a^	-	-	0.00^a^	-	-
**X5**		0.00	0.01	0.650	-0.01	0.07	0.913	0.04	0.04	0.311
**X6**		0.04	0.01	**0.001**	0.18	0.08	**0.018**	-0.04	0.04	0.353
**X7**		0.00	0.00	**<0.001**	-0.01	0.00	**0.001**	0.00	0.00	0.542

Note. X1: Regional location; X2: Informationization level; X3: Involvement in the family GP program

X4: Medical association participation; X5: Number of sick beds; X6: Number of employees

X7: Floor area of the buildings; DC = Demonstrating center, KC = Key center, SC = Standard center.

^a^ The parameter is redundant, so it is set as 0.

*significant at the 0.05 level (2-tailed).

^①^In the model of overall performance, the interaction (Year*X2, Year*X6, Year*X7) were significant (*P*<0.05).

^②^In the model of institutional management, the interaction (Year*X7) was significant (*P*<0.05).

Table 5 displayed the year, level of informationization system and family GP program were found to be the significant predictor variables in the model of basic medical services performance. In the model of CTM services performance were found the influencing factors of regional location, level of informationization system and medical association participation while there were statistical interaction between year and level of informationization system as well as year and floor area of the buildings. And in the model of comprehensive satisfaction performance, the year, level of informationization system and medical association participation were recognized as predictors, nevertheless, the interaction of year and level of informationization system should not be ignored.

**Table 5:** Analysis of influencing factors on basic medical services, CTM services and comprehensive satisfaction performance.

Independent variables		Basic medical services			CTM services^①^			Comprehensive satisfaction^②^		
		β	SE	*P* *	β	SE	*P* *	β	SE	*P* *
**Intercept**		117.65	8.98	**<0.001**	64.17	4.43	**<0.001**	66.37	5.61	**<0.001**
**Year**	**2013y**	49.79	2.86	**<0.001**	5.85	6.29	0.352	32.83	4.53	**<0.001**
	**2012y**	47.29	3.49	**<0.001**	-2.75	9.51	0.773	0.12	5.19	0.981
	**2011y**	0.00^a^	-	-	0.00^a^	-	-	0.00^a^	-	-
**X1**	**Urban division**	2.86	3.81	0.453	6.18	2.14	**0.004**	3.40	1.99	0.088
	**Suburban district**	-1.11	3.31	0.738	6.07	1.77	**0.001**	-0.78	1.53	0.611
	**Rural area**	0.00^a^	-	-	0.00^a^	-	-	0.00^a^	-	-
**X2**	**High**	26.35	5.14	**<0.001**	17.38	4.34	**<0.001**	23.74	6.04	**<0.001**
	**Medium**	21.75	4.53	**<0.001**	19.58	3.53	**<0.001**	13.38	5.05	**0.008**
	**Low**	0.00^a^	-	-	0.00^a^	-	-	0.00^a^	-	-
**X3**	**DC**	3.81	5.33	0.475	-0.41	3.33	0.903	0.10	2.91	0.972
	**KC**	7.38	4.39	0.093	2.68	2.22	0.227	1.67	2.57	0.515
	**SC**	0.00^a^	-	-	0.00^a^	-	-	0.00^a^	-	-
	**Yes**	13.80	3.95	**<0.001**	0.34	3.52	0.922	-0.10	2.78	0.973
	**No**	0.00^a^	-	-	0.00^a^	-	-	0.00^a^	-	-
**X4**	**Yes**	-2.69	3.43	0.432	-6.13	1.77	**0.001**	-6.01	1.65	**<0.001**
	**No**	0.00^a^	-	-	0.00^a^	-	-	0.00^a^	-	-
**X5**		0.03	0.04	0.534	0.02	0.02	0.396	0.02	0.02	0.314
**X6**		0.00	0.04	0.934	0.01	0.02	0.462	-0.02	0.02	0.475
**X7**		0.00	0.00	0.641	0.00	0.00	0.891	0.00	0.00	0.198

Note. X1: Regional location; X2: Informationization level; X3: Involvement in the family GP program

X4: Medical association participation; X5: Number of sick beds; X6: Number of employees

X7: Floor area of the buildings; DC = Demonstrating center, KC = Key center, SC = Standard center.

^a^ The parameter is redundant, so it is set as 0.

*significant at the 0.05 level (2-tailed).

^①^In the model of CTM services, the interaction (Year*X2, Year*X7) were significant (*P*<0.05).

^②^In the model of comprehensive satisfaction, the interaction (Year*X2) was significant (*P*<0.05).

## Discussion

In China, the state-owned health institutions obtain their funding from a variety of sources, and CHSCs rely mainly on the local governmental support plus running-profit, while other public hospitals are more likely to be self-sufficient, gaining their profits from prescription drugs [18]. Therefore, the configuration for a CHSC is in accordance with the local economy, size of the area and local population. In Pudong, the CHSCs account for up to 25% of all in the city of Shanghai, the number higher than that in any other district. And their distribution is relatively uniform in different types of areas. With the population of immigrants increasing rapidly, however, the allocation of one CHSC for a population of 100,000 residents is still a challenge. In respect of sociological characteristics, there are differences between CHSCs not only in area size and informationization construction, but in investment for health-care reforms. Some have joined the GP family program and medical association program, even though their impact on holistic performance hasn’t been confirmed. In the current investigation, therefore, we adopted a comprehensive evaluation method, which was revised based on the national framework and featured the local indicators, thus spotting the performance trend of CHSCs in Pudong from 2011 to 2013, as well as its major influencing factors.

The data showed that the differences in overall performance were significant among the CHSCs, whether cross- or vertical-comparable. In general, these differences could be attributed to the sociological characteristics of the CHSC and the implementation of the health care policies. Most of those which are located in the high-density populated subdivisions were the example centers for the scoring of better performance in the country. On the other hand, those which are categorized as rural areas or suburban districts ranked lower, and they seemed to be less competent at providing sufficient health care service and capable of improving their working environment.

However, almost all the centers showed some improvement over the period of the investigation, even though the gap was large among them in 2011. The differences were observed to be gradually narrowed, which means that a growing number of the centers progressed towards the good and excellent ranks. Those in the rural areas still have room for improvement if some interventions can be implemented. But we have to admit that the reform in health care, as in the case of others in the country, is an evolutionary process, and it takes time to exert a profound impact. We presume that the effects of the latest reforms will be more productive if we allow them more time.

In terms of the various aspects of performance in the CHSCs, the data revealed that both higher scores were observed in the services of public health and CTM than in those of the institutional management and basic medical services which we believe could downgrade overall performance. The high achievement in public health services should be accounted for by the new policies for public health service equality, which guaranteed the funding for each resident, while the improved CTM services were ascribed to the national CTM services pilot program. A number of the centers targeted the framework of Shanghai-based standardized CTM services so that they provided various CTM care items for outpatients.

It was the institutional management that assured the rising tendency in overall performance during the period of three years. The health administrative departments paid more attention to the allocation of health resources and the cultivation of medical talents. In order to realize the aim that everyone will have access to basic medical and health services in 2020, China has enforced a series of specific health policies, whose effect could be reflected in the current investigation, i.e. the basic medical services experienced a rapid promotion. Similarly, the comprehensive satisfaction improved significantly. We have every reason to believe that the better performance in the basic medical services upgraded the comprehensive satisfaction level, and that high quality and accessibility of basic medical services will assist in the development of CHS.

As to the sociological factors of the CHSCs, the informationization level was shown to be the most significant influencing factor to the overall or dimension-specific performance level based on the analysis. The annually increasing scores of informationization showed in the survey indicated that the level of the informationization system had been gradually improved and strengthened. Obviously, it became a crucial element in performance. Of course, there was no denying the timing-effect to the informationization system. Therefore it is imperative to build a consistent, interactive and integrated primary service network of the CHSCs in the age of information [19]. The hospital connection network, as a key approach to improving the capability and efficacy of medical services, can also establish and improve the two-way referral of patients.

The initiative policy of medical association participation was another factor that exerted a significant impact on overall performance, as well as on the performance of institutional management, public health service, CTM services and comprehensive satisfaction. Regrettably, we found the CHSCs which involved with the program of medical association presented lower scores on overall performance and comprehensive satisfaction than those which did not. This might have been caused by the patients’ restricted choices of hospitals and the shortage of cooperation and assistance from the upper-level hospitals in referral process. However, we shouldn’t deny the effectiveness of the policy when such evidence has been found in other developed countries [20,21]. It is essential to reflect on our institutional management, especially benefit or profit distribution so as to make the initiative adoptable here in China.

The family GP program as another initiative policy showed a positive impact on overall performance, institutional management and basic medical services, where medical association participation did not have the same effect. This was in conformity with the role of the family GP playing in the basic medical service during the reforms. Consequently, the family GP as a link, in these pilot CHSCs the patient-physician relationship was improved effectively, so was the patients’ cooperation with the doctors [22].

In addition, we came to notice that the regional location had a positive relation with overall performance, institutional management, CTM services and comprehensive satisfaction, which implied that economic inequalities between the urban and rural areas can have a great impact on CHS performance. In general, a CHSC makes more average income in treating wealthier patients in a better-developed area with a larger population, because public health service has a close relation with its population. The southern towns in Pudong, mainly lying in the rural areas and suburban districts and covering larger areas with a lower population density than the northern urban ones, have their residents who are less likely to afford regular medical treatment, making these centers hard to improve. This should urge the local government to take action to compensate for the shortages of certain underdeveloped areas.

On the contrary, although the number of sick beds and size of building area showed a little impact on the overall performance and institutional management performance, as both variables relate to the size of the CHSC, we tentatively concluded that the size would not affect the overall performance. Therefore, it may not be suitable to develop a center by extending its size without limit.

## Conclusions

The current investigation showed that the overall performance of the CHSCs in Pudong of Shanghai has improved from 2011 to 2013, but there is still room for further improvement, especially in institutional management and basic medical service. Furthermore, the level of the informationization system, medical association participation, regional location and the family GP program were the main contributing factors to the overall performance, rather than the size of the center. With further health care reforms, the level of the informationization system, family GP program and regional location played a positive role in improving performance, while the medical association program didn’t show a significant beneficial effect. Above all, our study suggests that the administrative departments on health should tackle the inequality among the CHSCs, providing the underdeveloped with imperative support, establishing and reinforcing the informationization system, expanding the coverage of the family GP program, and promoting the medical association program.

## Supporting Information

S1 File Comprehensive satisfaction survey of patients of community health services centers in Pudong New Area. (DOC)

S2 File Informed Consent of the patients (Study on performance evaluation of community health services centers in Pudong new district). (DOC)

S3 File Satisfaction survey of medical personnel of community health services centers in Pudong New Area. (DOCX)

S4 File Informed Consent of medical personnel (Study on performance evaluation of community health services centers in Pudong new district). (DOCX)

S5 File The satisfaction survey of general practitioner service in Pudong New District. (DOC)

S6 File The fairness and privacy statement of research on performance evaluation of community health services in Pudong New District. (DOC)

S7 File Review comment on the ethics application to the research on performance evaluation of community health services in Pudong New Area. (DOCX)

S8 File The index of performance assessment of community health service centers in Pudong new area. (DOCX)
