# Supplementary material for: Enhanced Performance of Community Health Service Centers during Medical Reforms in Pudong New District of Shanghai, China: A Longitudinal Survey
Source: PLoS One. 2015 May 7;10(5):e0125469. doi: 10.1371/journal.pone.0125469 (PMC4423872; doi:10.1371/journal.pone.0125469)
Supplement: S1 File — (DOC) [file pone.0125469.s001.doc]

**Comprehensive satisfaction survey of patients of community health services centers in Pudong New Area**

Sex： Age： Permanent residents：□Yes（□Shanghai □Non-Shanghai） □No

1. Is it convenient to this community health services center to see the doctor？

(1)very convenient (2)convenient (3)just so so (4)inconvenient (5)Very inconvenient

2. Are you satisfied with Queuing time in community health services centers?

(1)very satisfied (2)satisfied (3)just so so (4)unsatisfied (5)very unsatisfied

Reasons of dissatisfaction：

3. Are you satisfied with the medical environment?

(1)very satisfied (2)satisfied (3)just so so (4)unsatisfied (5)very unsatisfied

Reasons of dissatisfaction：

4. Are you satisfied with the facilities here?

(1)very satisfied (2)satisfied (3)just so so (4)unsatisfied (5)very unsatisfied

Reasons of dissatisfaction：

5. Are you satisfied with the service attitude of the medical staffs?

(1)very satisfied (2)satisfied (3)just so so (4)unsatisfied (5)very unsatisfied

Reasons of dissatisfaction：

6. Are you satisfied with the interpretation, communication and service of the doctors?

(1)very satisfied (2)satisfied (3)just so so (4)unsatisfied (5)very unsatisfied

Reasons of dissatisfaction：

7. Are you satisfied with the cost of seeing a doctor?

(1)very satisfied (2)satisfied (3)just so so (4)unsatisfied (5)very unsatisfied

Reasons of dissatisfaction：

8. Are you satisfied with the doctors’ medical technology here?

(1)very satisfied (2)satisfied (3)just so so (4)unsatisfied (5)very unsatisfied

Reasons of dissatisfaction：

9. Are you satisfied with medical treatment here?

(1)very satisfied (2)satisfied (3)just so so (4)unsatisfied (5)very unsatisfied

Reasons of dissatisfaction：

10. Are you satisfied with service effectiveness?

(1)very satisfied (2)satisfied (3)just so so (4)unsatisfied (5)very unsatisfied

Reasons of dissatisfaction：

**Summary：**

|  | very satisfied | satisfied | just so so | unsatisfied | very unsatisfied |
| --- | --- | --- | --- | --- | --- |
| number |  |  |  |  |  |
| total |  | |  | | |
| Satisfaction rate (%) | | |  | | |

P.S. Satisfaction rate =①/（①+②）×100

Investigator signature： date：
