# Supplementary material for: Enhanced Performance of Community Health Service Centers during Medical Reforms in Pudong New District of Shanghai, China: A Longitudinal Survey
Source: PLoS One. 2015 May 7;10(5):e0125469. doi: 10.1371/journal.pone.0125469 (PMC4423872; doi:10.1371/journal.pone.0125469)
Supplement: S2 File — (DOC) [file pone.0125469.s002.doc]

**Informed Consent of the patients**

**Study on performance evaluation of community health services centers in Pudong new district**

Dear Mr./Mrs. :

We are making the performance assessment of community health service organization in Pudong New district. Now we invite you to joining in our project, expressing the comprehensive satisfaction of the community health service centers you have ever visited, which include the access to centers, medical environment, medical facilities, the attitudes of service, service technologies, the cost and treatment effect, etc. It is important to help us deeply understand the service of community health service centers and find the problems timely, which will be of significance to deepen the reform of community health service.

We abide by the Statistics Law and you can fill the questionnaire anonymously, which will cost you 5 minutes. Moreover, all your answers will be kept strictly confidential.

Thank you very much for your support and cooperation!

Best wish to you and have a happy life!

If you has been informed and agreed to the above survey information, please sign your name：**____________________**

Shanghai Pudong Institute for Health Development

Research group of performance evaluation of community health services in Pudong new district

Date：
