# Supplementary material for: Enhanced Performance of Community Health Service Centers during Medical Reforms in Pudong New District of Shanghai, China: A Longitudinal Survey
Source: PLoS One. 2015 May 7;10(5):e0125469. doi: 10.1371/journal.pone.0125469 (PMC4423872; doi:10.1371/journal.pone.0125469)
Supplement: S3 File — (DOCX) [file pone.0125469.s003.docx]

**Satisfaction survey of medical personnel of community health services centers in Pudong New Area**

Sex: Age:

1. Are you satisfied with your working environment (e.g. software and hard ware environment, working and studying environment, etc.)?

(1) very satisfied (2)satisfied (3)just so so (4)unsatisfied (5)very unsatisfied

The reason why you are unsatisfied:

2. Are you satisfied with the community health services centers’ internal management (e.g. regulation, staff motivation, etc.)?

(1) very satisfied (2)satisfied (3)just so so (4)unsatisfied (5)very unsatisfied

The reason why you are unsatisfied:

3. Are you satisfied with the remuneration (e.g. performance related salary, etc.)?

(1) very satisfied (2)satisfied (3)just so so (4)unsatisfied (5)very unsatisfied

The reason why you are unsatisfied:

4. Are you satisfied with the community health services centers’ training (e.g. position qualification training, advanced studies, etc.)?

(1) very satisfied (2)satisfied (3)just so so (4)unsatisfied (5)very unsatisfied

The reason why you are unsatisfied:

5. Are you satisfied with the technical appraisal and employment (e.g. standard, process, etc.)?

(1) very satisfied (2)satisfied (3)just so so (4)unsatisfied (5)very unsatisfied

The reason why you are unsatisfied:

6. Are you satisfied with the personal development?

(1) very satisfied (2)satisfied (3)just so so (4)unsatisfied (5)very unsatisfied

The reason why you are unsatisfied:

**Summary：**

|  | very satisfied | satisfied | just so so | unsatisfied | very unsatisfied |
| --- | --- | --- | --- | --- | --- |
| number |  |  |  |  |  |
| total |  | |  | | |
| Satisfaction rate (%) | | |  | | |

P.S. Satisfaction rate =①/（①+②）×100

Investigator signature： date：
