# Supplementary material for: Enhanced Performance of Community Health Service Centers during Medical Reforms in Pudong New District of Shanghai, China: A Longitudinal Survey
Source: PLoS One. 2015 May 7;10(5):e0125469. doi: 10.1371/journal.pone.0125469 (PMC4423872; doi:10.1371/journal.pone.0125469)
Supplement: S4 File — (DOCX) [file pone.0125469.s004.docx]

**Informed Consent of medical personnel**

**Study on performance evaluation of community health services centers in Pudong new district**

To whom it may concerned,

We are the now evaluating the performance of community health services centers (CHSCs) and need your cooperation. This questionnaire is to assess the staff comprehensive satisfaction of CHSCs, including six aspects of working environment, internal management, remuneration, training, technical appraisal and employment, and personal development. It may help us find the problems of the CHSC reform, meaningful to the improvement of CHSC ability.

We strictly conform to the Low of Statistics. The survey is all anonymous and confidential. Hope you can give us 5 minutes.

Thank you for your time!

If you has been informed and agreed to the above information, please sign your name here:

Shanghai Pudong Institue for Health development

Research group of performance evaluation of community health services centers in Pudong
