# Supplementary material for: Enhanced Performance of Community Health Service Centers during Medical Reforms in Pudong New District of Shanghai, China: A Longitudinal Survey
Source: PLoS One. 2015 May 7;10(5):e0125469. doi: 10.1371/journal.pone.0125469 (PMC4423872; doi:10.1371/journal.pone.0125469)
Supplement: S5 File — (DOC) [file pone.0125469.s005.doc]

**The satisfaction survey of general practitioner service in Pudong New District**

Organization：  Community Health service Center

| Respondents’ Information | | | | follow-up result | |
| --- | --- | --- | --- | --- | --- |
| No. | Name | Phone number | Committee | ① | ② |
| 1 |  |  |  |  |  |
| 2 |  |  |  |  |  |
| 3 |  |  |  |  |  |
| 4 |  |  |  |  |  |
| 5 |  |  |  |  |  |
| 6 |  |  |  |  |  |
| 7 |  |  |  |  |  |
| 8 |  |  |  |  |  |
| 9 |  |  |  |  |  |
| 10 |  |  |  |  |  |

Attention：

1. Please collect at least 10 customers and fill in the blank with A、B or C.

2. Content of telephone follow-up：

① Do you know the name of your family doctor? A.Yes B.No C.Not clear

② Do you think family doctor is necessary? A.Yes B.No experience C.No

**Summary：**

|  | ① | ② |
| --- | --- | --- |
| A |  |  |
| B |  |  |
| C |  |  |

Expert Sign： Follow-up time：
