# Supplementary material for: Enhanced Performance of Community Health Service Centers during Medical Reforms in Pudong New District of Shanghai, China: A Longitudinal Survey
Source: PLoS One. 2015 May 7;10(5):e0125469. doi: 10.1371/journal.pone.0125469 (PMC4423872; doi:10.1371/journal.pone.0125469)
Supplement: S6 File — (DOC) [file pone.0125469.s006.doc]

**The fairness and privacy statement of research on performance evaluation of community health services in Pudong New District**

I declare that I will consciously abide by the following criteria:

1. I will execute the program strictly, reflect the fact objectively and evaluate independently without the outside influence.

2. In case of the fairness of assessment, I will report to the principal ahead of time in order to take corresponding measures to ensure the impartiality of the assessment, such as：

(1) There is some certain relationship of economics benefits or other with appraisal institution；

(2) It is used to working in the appraisal institutions in recent two years；

(3) Other acts may affect the impartiality in the process.

3. I will strictly abide by the confidentiality provisions and shall divulge to third person about the information that the center provides and the evaluation without the permission of the center.

4. I promise not to damage the reputation of the center, or the interests of the assessed party.

If there is any violation of the rules, and cause complaints/economic compensation, I will bear the corresponding legal responsibility.

Confirmed by Offeree：

Date：
