# Supplementary material for: Enhanced Performance of Community Health Service Centers during Medical Reforms in Pudong New District of Shanghai, China: A Longitudinal Survey
Source: PLoS One. 2015 May 7;10(5):e0125469. doi: 10.1371/journal.pone.0125469 (PMC4423872; doi:10.1371/journal.pone.0125469)
Supplement: S7 File — (DOCX) [file pone.0125469.s007.docx]

**Review comment on the ethics application to the research on performance evaluation of community health services in Pudong New Area**

Xiao-ming Sun, Yan-ting Li, Shan-shan Liu, etc.:

We have received your ethics application about implementation performance evaluation of community health services in Pudong New Area. After checking the information submitted, the academic ethics committee of Shanghai Pudong institute for health development thought: Firstly, the community health service organizations being investigated are the affiliated units of health and family planning commission of Pudong new area, the relevant data refer to the institutes situation and involve no privacy records of the patients. Secondly, before the satisfaction survey of the patients and professional medical workers, they will sign the informed consent and are surveyed anonymously. Thirdly, the acquired data are only used for research and analyzed by blind methods, organization or person name being not been showed.
 Based on the above, the academic ethics committee agree that the program team have considered the relevant ethical issues to carry out the investigation, so the additional conference review are not needed.

Chairman of the academic ethics committee:

(Authorized Signatory)

The Academic ethics committee of Shanghai Pudong institute for health development

  July 20, 2011
