# Supplementary material for: Enhanced Performance of Community Health Service Centers during Medical Reforms in Pudong New District of Shanghai, China: A Longitudinal Survey
Source: PLoS One. 2015 May 7;10(5):e0125469. doi: 10.1371/journal.pone.0125469 (PMC4423872; doi:10.1371/journal.pone.0125469)
Supplement: S8 File — (DOCX) [file pone.0125469.s008.docx]

The index of performance assessment of community health service centers in Pudong new area

| The first class indicator | The second class indicator | The third class indicator | Weight | Scoring criteria |
| --- | --- | --- | --- | --- |
| 1. Institutional management  (410 points) | 1.1 Institutional environment  (18 points) | 1.Office layout | 10 | 2 points per item |
|  |  | 2.Service environment | 8 |  |
|  | 1.2 Human resource management  (14 points) | 1.Staff allocation | 8 | 4 points per item |
|  |  | 2.Staff performance appraisal | 6 | 3 points per item |
|  | 1.3 Continuing education  (14 points) | 1.Talent training | 9 | 3 points per item |
|  |  | 2.General practitioner training | 3 |  |
|  |  | 3.Joining in the health personnel training plan of the bureau | 2 | 2 points per item |
|  | 1.4 Academic research  (15 points) | 1.Science research and education management | 5 | 5 points≥90% of the average of the whole district or reduction per 1% cut down 0.2 points |
|  |  | 2.The scientific research project | 5 |  |
|  |  | 3.The paper presented | 5 |  |
|  | 1.5Financial and assets management  (28 points) | 1.Accounting | 1.5 | 0.5 points per item |
|  |  | 2.The budget management | 1 |  |
|  |  | 3.Asset management | 5 |  |
|  |  | 4.Income and expense management | 14 |  |
|  |  | 5.Purchasing and supply management | 2 |  |
|  |  | 6.Bill management | 4.5 |  |
|  | 1.6Drugs management  (18 points) | 1.General drugs management | 7 | 1.5 points per item |
|  |  | 2.Special drugs management | 3 | 1 points per item |
|  |  | 3.The proportion of drugs | 8 | 2 points per item |
|  | 1.7▲Rural Health management  (12 points) | 1.Village clinic management | 6 | 5 points per item |
|  |  | 2.Rural doctors management | 6 |  |
|  | 1.8▲The new rural cooperative medical care management  (18 points) | 1.Organizational guarantee | 2 |  |
|  |  | 2.Cost control | 15 |  |
|  |  | 3.Information management | 1 |  |
|  | 1.9 Construction of spiritual civilization  (28 points) | 1.System construction | 5 | 0.5 points per item |
|  |  | 2.Medical ethics | 6 | 2 points per item |
|  |  | 3.Propaganda contribution | 6 |  |
|  |  | 4.Civilization and window services | 8 |  |
|  |  | 5.Information disclosure | 3 | 1 points per item |
|  | 1.10Information management  (10 points) | 1.Information sharing and data security maintenance | 7 | 0.5 points per item |
|  |  | 2.Informatization construction | 3 |  |
|  | 1.11Service mode  (118 points) | 1.Community participation | 8 | 2 points per item |
|  |  | 2.Collaborative services | 4 |  |
|  |  | 3.Active services | 6 |  |
|  |  | 4.General practitioner family responsibility system | 100 |  |
|  | 1.12Operating efficiency  (100 points) | 1.Employee contribution per capita | 25 | The baseline for regional average for 16, rise per 1% increase 0.5 points |
|  |  | 2.The contribution to government spending | 25 |  |
|  |  | 3.The contribution of special equipment | 20 | The baseline for regional average for 14, rise per 1% increase 0.2 points |
|  |  | 4.Non-medical costs for the service of health care | 20 |  |
|  |  | 5. Health supplies cost per capita | 10 | 5 points per item |
|  | 1.13The safety management | 1.Medical accident | Punitive | Cut down 100 points per case |
|  |  | 2.Safety accidents |  |  |
|  |  | 3.Agency compensation |  |  |
|  | 1.14The rectification follow-up  (17 points) | The rectification effect and material preparation | 17 | 6 points per item, 1~5 points partly |
| 2. Public health service  (350 points) | Public health management  (350 points) | 1.Health care of child 0-6 years old | 33 | According to the annual appraisal results of public health line |
|  |  | 2.Vaccination | 42.6 |  |
|  |  | 3.School (childcare facilities) health care | 22.2 |  |
|  |  | 4.Women health care | 34 |  |
|  |  | 5.Occupational health | 3.8 |  |
|  |  | 6.The elderly care, eye disease prevention and control | 13.1 |  |
|  |  | 7.Chronic disease prevention and control | 40 |  |
|  |  | 8.Mental health | 19.2 |  |
|  |  | 9.Infectious diseases and emergency emergency disposal | 37.2 |  |
|  |  | 10.Health supervision and assist | 19.2 |  |
|  |  | 11.Management of disease and its related factors | 14 |  |
|  |  | 12.Health education | 19.2 |  |
|  |  | 13.Vital statistics | 2.5 |  |
|  |  | 14.Elderly medical | 30 |  |
|  |  | 15.Health records | 20 |  |
|  |  | 16.Major responsibility event of public health | Punitive | Cut down 100 points per case |
| 3. Basic medical service  (240 points) | 3.1The medical service ability  (56 points) | 1.Outpatient service and emergency services | 50 | The baseline for regional average for 16, rise per 1% increase 0.2 points or decrease |
|  |  | 2Bed utilization rate | 6 | Full points ≥90% of the average or reduction per 1% cut down 0.5 points |
|  | 3.2Medical quality control(24 points) | The qualification rate of medical documents writing | 24 |  |
|  | 3.3Nursing quality control  (15 points) | 1.Transfusion management | 5 |  |
|  |  | 2.Year-end assessment result | 10 |  |
|  | 3.4Clinical laboratory quality control  (10 points) | 1.The quality of clinical laboratory | 5 |  |
|  |  | 2.Year-end appraisal result | 5 |  |
|  | 3.5Radiological examination quality control  (10 points) | 1.Radiological examination quality | 5 |  |
|  |  | 2.Year-end appraisal result | 5 |  |
|  | 3.6Electrocardiogram (Ecg) and B-ultrasonic wave quality control(10 points) | 1.Electrocardiogram (Ecg) quality | 2.5 |  |
|  |  | 2.B-ultrasonic examination quality | 2.5 |  |
|  |  | 3.Year-end appraisal result | 5 |  |
|  | 3.7Nosocomial infection management  (11 points) | 1.The incidence of nosocomial infection | 7 | Full points ≤7% or increase per 1% cut down 0.5 points |
|  |  | 2.Nosocomial infection management system | 4 | 1 points per item |
|  | 3.8The rational use of drugs  (20 points) | 1.The proportion of antibiotics application prescription | 10 | Full points ≤20% or increase per 1% cut down 1 point |
|  |  | 2.The proportion of intravenous drug prescription | 10 | Full points ≤15% or increase per 1% cut down 1 point |
|  | 3.9Rehabilitation services  (30 points) | 1.Service facility | 10 | 2 points per item |
|  |  | 2.Services provide | 14 |  |
|  |  | 3.Services management | 6 |  |
|  | 3.10Health care costs(10 points) | Monthly diagnosis and treatment of outpatient expense | 10 | The baseline for regional average for 6, descend per 1% increase 0.15 points |
|  | 3.11Management of health treatment insurance  (35 points) | 1.Total amount control | 10 | The baseline for regional average for 6, descend per 1% increase 1 points |
|  |  | 2.Medicine amount control | 10 |  |
|  |  | 3.Total to share | Punitive | increase per 1% cut down 1 point, subtract 10 points the most |
|  |  | 4.Each outpatient average cost | 5 | Full points ≤ the average or rise per 1% cut down 0.5 points |
|  |  | 5.Outpatient follow-up rate | 5 |  |
|  |  | 6.The daily average cost in hospital | 5 |  |
|  | 3.12Management by law  (9 points) | 1.Physician practice place | 3 | Cut down all points per case |
|  |  | 2.Registered subject | 3 |  |
|  |  | 3.Licensed to practice medicine | 3 |  |
| 4. Chinese traditional medicine service  (100 points) | 4.1The service number  (40 points) | 1.TCM outpatient quantity proportion | 9 | According to the annual appraisal results of Chinese traditional medicine line |
|  |  | 2.The proportion of number of non-drug therapy of TCM | 9 |  |
|  |  | 3.The proportion of TCM prescription number | 9 |  |
|  |  | 4.Constitution of TCM identification | 1.5 |  |
|  |  | 5.TCM prevention and control of diabetes | 1.5 |  |
|  |  | 6.TCM prevention and treatment of hypertension | 1.5 |  |
|  |  | 7.TCM prevention and treatment of chronic hepatitis B | 0.5 |  |
|  |  | 8.TCM health education | 0.5 |  |
|  |  | 9.Regulation of TCM health care institutions | 0.5 |  |
|  |  | 10.TCM health care during pregnancy | 1 |  |
|  |  | 11.The postpartum TCM health care | 1 |  |
|  |  | 12.Pregnant women school education of TCM | 0.5 |  |
|  |  | 13.Children constitution of TCM identification | 1 |  |
|  |  | 14.Children health propaganda and education of TCM | 0.5 |  |
|  |  | 15.TCM health care students myopia | 1.5 |  |
|  |  | 16.TCM intervention of cognitive impairment in the elderly | 1.5 |  |
|  | 4.2The quality of service(60 points) | The quality control of Chinese medicine service | 60 |  |
| 5. Comprehensive satisfaction  (100 points) | 5.1The comprehensive satisfaction of patients | | 60 | Full points ≥90 or reduction per 1% cut down 1 point |
|  | 5.2 The satisfaction of health technical personnel | | 40 |  |
|  | 5.3Number of complaints | | Punitive | Cut down 1 point per case |
| Total | | | 1200 |  |
